# Exploring the Role of Fatty Acid Supplementation in Persons with Alzheimer’s Disease: A Scoping Review

**DOI:** 10.1002/alz.090004

**Published:** 2025-01-09

**Authors:** Mahi Basra, Saajan Patel, Kriya Shah, Joshua Caballero

**Affiliations:** ^1^ Nova Southeastern Univeristy, Kiran C. Patel College of Osteopathic Medicine, Clearwater, FL USA; ^2^ Nova Southeastern University, Dr. Kiran C. Patel College of Osteopathic Medicine ‐ TBR, Clearwater, FL USA; ^3^ Nova Southeastern University, Kiran C. Patel College of Osteopathic Medicine, Davie, FL USA; ^4^ University of Georgia, College of Pharmacy, Athens, GA USA

## Abstract

**Background:**

Lipids are key modulators in the pathogenesis of Alzheimer’s disease (AD). Dysregulation of lipid homeostasis may disrupt the blood brain barrier, alter myelination, disturb cellular signaling and cause abnormal processing of the amyloid precursor protein. The purpose of this scoping review was to evaluate fatty acid supplementation in patients with AD.

**Method:**

A literature search was completed using PubMed, EMBASE and Medline identifying peer‐reviewed articles published between January 2015 and November 2023. Randomized controlled trials, correlational studies, clinical trials, and case studies were considered, while meta‐analyses, systematic reviews, scoping reviews, animal studies, and non‐English literature were excluded. The search strategy involved key terms such as “Alzheimer,” “Alzheimer’s Disease,” “AD,” “neuroinflammation,” “fatty acids,” “fat,” and “lipids.” The PRISMA guidelines guided the inclusion/exclusion process.

**Result:**

Of the 392 articles initially identified, five met inclusion criteria and were evaluated. Omega‐3 polyunsaturated fatty acid (i.e., fish oil), docosahexaenoic/eicosapentaenoic acid (DEA/EPA), medium‐chain triglycerides (MCT), and a combination product made of docosahexaenoic acid (DHA), EPA, uridine monophosphate, choline, phospholipids, selenium, B/C/E vitamins, and folic acid (Souvenaid^®^) were used in people with AD. A small study using fish oil (0.45 g of EPA with 1 g of DHA) over one year, showed decreased levels of carbonyl groups and hydroperoxides, but clinical effects were not evaluated. A study using DEA/EPA supplementation showed a decrease in fatty acid metabolism products and prevented deterioration in MMSE scores after 6 months. Additionally, the effect of MCT on cognitive ability in 53 patients with mild to moderate AD suggested APOE4‐/‐ patients with mild to moderate AD may have benefited from the MCT intervention with improved cognitive ability (ADAS‐Cog score) compared to placebo. Other studies evaluating Souvenaid^®^ in combination with acetylcholinesterase inhibitors suggested improvements in behavioral tasks, cognitive function, and increase in hippocampal volume.

**Conclusion:**

Preliminary trials suggested mild improvements in persons with AD with fatty acid supplementation. Souvenaid^®^ and MCTs seem promising, highlighting potential clinical benefits. However, there is a need for additional extensive studies to validate these findings and establish clinical significance in AD.

